# A176 ASSOCIATIONS OF ANTIBIOTICS, HORMONAL THERAPIES, ORAL CONTRACEPTIVES, AND LONG-TERM NSAIDS WITH INFLAMMATORY BOWEL DISEASE: RESULTS FROM THE PROSPECTIVE URBAN RURAL EPIDEMIOLOGY (PURE) STUDY

**DOI:** 10.1093/jcag/gwac036.176

**Published:** 2023-03-07

**Authors:** C Pray, N Narula, E C Wong, J K Marshall, S Rangarajan, S Islam, A Bahonar, K F Alhabib, A Kontsevaya, F Ariffin, H U Co, W Al Sharief, A Szuba, A Wielgosz, M L Diaz, R Yusuf, L Kruger, B Soman, Y Li, C Wang, L Yin, M Erkin, F Lanas, K Davletov, A Rosengren, P Lopez-Jaramillo, R Khatib, A Oguz, R Iqbal, K Yeates, Á Avezum, W Reinisch, P Moayyedi, S Yusuf

**Affiliations:** 1 Department of Medicine (Division of Gastroenterology) and Farncombe Family Digestive Health Research Institute, McMaster University; 2 Population Health Research Institute, McMaster University and Hamilton Health Sciences; 3 McMaster University and Hamilton Health Sciences, Population Health Research Institute, Hamilton, Canada; 4 Isfahan Cardiovascular Research Center, Isfahan, Iran, Islamic Republic Of; 5 King Fahad Cardiac Center, King Saud Medical City, Saudi Arabia; 6 National research center for therapy and preventive medicine, Moscow, Russian Federation; 7 Faculty of Medicine UiTM, Selangor, Malaysia; 8 University of the Philippines College of Medicine, Ermita, Philippines; 9 Family Medicine Department, Medical Education & Research Department in Dubai Health Authority (DHA), Oud Metha-Dubai, United Arab Emirates; 10 Wroclaw Medical University, Wroclaw, Poland; 11 University of Ottawa Heart Institute, Ottawa, Canada; 12 Estudios Clínicos Latino América, Rosario, Argentina; 13 Independent University, Bangladesh, Bashundhara , Bangladesh; 14 Africa Unit for Transdisciplinary Health Research , North West University, Potchefstroom, South Africa; 15 Sree Chitra Tirunal Institute for Medical Sciences and Technology, Trivandrum, India; 16 Medical Research & Biometrics Center, Peking Union Medical College & Chinese Academy of Medical Sciences, Beijing, China; 17 Kyrgyz State Medical Academy, Bishkek, Kyrgyzstan; 18 Universidad de La Frontera, Temuco, Chile; 19 Al-Farabi Kazakh National University, Almaty, Kazakhstan; 20 Sahlgrenska Academy, University of Gothenburg , Gothenburg , Sweden; 21 Masira Research Institute, Universidad de Santander , Bucaramanga, Colombia; 22 Institute of Community and Public Health, Birzeit University,, Birzeit, Palestinian, State of; 23 Internal Medicine, Istanbul Medeniyet University,, Istanbul, -; 24 Department of Community Health Sciences, Aga Khan University, Karachi City, Pakistan; 25 Department of Medicine, Queen's University, Kingston, Canada; 26 International Research Center, Hospital Alemão Oswaldo Cruz, São Paulo, Brazil; 27 Division of Gastroenterology and Hepatology, Medical University of Vienna, Vienna, Austria

## Abstract

**Background:**

The pathogenesis of inflammatory bowel disease (IBD) which includes Crohn’s disease (CD) and ulcerative colitis (UC), is believed to involve activation of the intestinal immune system in response to the gut microbiome among genetically susceptible hosts. IBD has been historically regarded as a disease of developed nations, though in the past two decades there has been a reported shift in the epidemiological pattern of disease. High-income nations with known high prevalence of disease are seeing a stabilization of incident cases, while a rapid rise of incident IBD is being observed in developing nations. This suggests that environmental exposures may play a role in mediating the risk of developing IBD. The potential environmental determinants of IBD across various regions is vast, though medications have been increasingly recognized as one broad category of risk factors.

**Purpose:**

Several medications have been considered to contribute to the etiology of IBD. This study assessed the association between medication use and risk of developing IBD using the Prospective Urban Rural Epidemiology (PURE) cohort.

**Method:**

This was a prospective cohort study of 133,137 individuals between the ages of 20-80 from 24 countries. Country-specific validated questionnaires documented baseline and follow-up medication use. Participants were followed prospectively at least every 3 years. The main outcome was development of IBD, including CD and UC. Short-term (baseline but not follow-up use) and long-term use (baseline and subsequent follow-up use) was evaluated. Results are presented as adjusted odds ratios (aOR) with 95% confidence intervals (CI).

**Result(s):**

During the median follow-up of 11.0 years [interquartile range (IQR) 9.2-12.2], we recorded 571 incident cases of IBD (143 CD and 428 UC). Higher risk of incident IBD was associated with baseline antibiotic use [aOR: 2.81 (95% CI: 1.67-4.73), p=0.0001] and hormonal medication use [aOR: 4.43 (95% CI: 1.78-11.01), p=0.001]. Among females, previous or current oral contraceptive use was also associated with IBD development [aOR: 2.17 (95% CI: 1.70-2.77), p=5.02E-10]. NSAID users were also observed to have increased risk of IBD [aOR: 1.80 (95% CI: 1.23-2.64), p=0.002], which was driven by long-term users [aOR: 5.58 (95% CI: 2.26-13.80), p<0.001]. All significant results were consistent in direction for CD and UC with low heterogeneity.

**Conclusion(s):**

Antibiotics, hormonal medications, oral contraceptives, and long-term NSAID use were associated with increased odds of incident IBD after adjustment for covariates.

**Please acknowledge all funding agencies by checking the applicable boxes below:**

Other

**Please indicate your source of funding below:**

Salim Yusuf is supported by the Heart & Stroke Foundation/Marion W. Burke Chair in Cardiovascular Disease. The PURE Study is an investigator-initiated study funded by the Population Health Research Institute, the Canadian Institutes of Health Research (CIHR), Heart and Stroke Foundation of Ontario, support from CIHR’s Strategy for Patient Oriented Research (SPOR) through the Ontario SPOR Support Unit, as well as the Ontario Ministry of Health and Long-Term Care and through unrestricted grants from several pharmaceutical companies, with major contributions from AstraZeneca (Canada), Sanofi-Aventis (France and Canada), Boehringer Ingelheim (Germany and Canada), Servier, and GlaxoSmithkline, and additional contributions from Novartis and King Pharma and from various national or local organisations in participating countries; these include: Argentina: Fundacion ECLA; Bangladesh: Independent University, Bangladesh and Mitra and Associates; Brazil: Unilever Health Institute, Brazil; Canada: Public Health Agency of Canada and Champlain Cardiovascular Disease Prevention Network; Chile: Universidad de la Frontera; China: National Center for Cardiovascular Diseases; Colombia: Colciencias, grant number 6566-04-18062; India: Indian Council of Medical Research; Malaysia: Ministry of Science, Technology and Innovation of Malaysia, grant numbers 100 -IRDC/BIOTEK 16/6/21 (13/2007) and 07-05-IFN-BPH 010, Ministry of Higher Education of Malaysia grant number 600 -RMI/LRGS/5/3 (2/2011), Universiti Teknologi MARA, Universiti Kebangsaan Malaysia (UKM-Hejim-Komuniti-15-2010); occupied Palestinian territory: the UN Relief and Works Agency for Palestine Refugees in the Near East, occupied Palestinian territory; International Development Research Centre, Canada; Philippines: Philippine Council for Health Research & Development; Poland: Polish Ministry of Science and Higher Education grant number 290/W-PURE/2008/0, Wroclaw Medical University; Saudi Arabia: the Deanship of Scientific Research at King Saud University, Riyadh, Saudi Arabia (research group number RG -1436-013); South Africa: the North-West University, SANPAD (SA and Netherlands Programme for Alternative Development), National Research Foundation, Medical Research Council of SA, The SA Sugar Association (SASA), Faculty of Community and Health Sciences (UWC); Sweden: grants from the Swedish state under the Agreement concerning research and education of doctors; the Swedish Heart and Lung Foundation; the Swedish Research Council; the Swedish Council for Health, Working Life and Welfare, King Gustaf V’s and Queen Victoria Freemasons Foundation, AFA Insurance, Swedish Council for Working Life and Social Research, Swedish Research Council for Environment, Agricultural Sciences and Spatial Planning, grant from the Swedish State under the Läkar Utbildnings Avtalet agreement, and grant from the Västra Götaland Region; Turkey: Metabolic Syndrome Society, AstraZeneca, Turkey, Sanofi Aventis, Turkey; United Arab Emirates (UAE): Sheikh Hamdan Bin Rashid Al Maktoum Award For Medical Sciences and Dubai Health Authority, Dubai UAE.

**Disclosure of Interest:**

C. Pray: None Declared, N. Narula Grant / Research support from: Neeraj Narula holds a McMaster University Department of Medicine Internal Career Award. Neeraj Narula has received honoraria from Janssen, Abbvie, Takeda, Pfizer, Merck, and Ferring, E. C. Wong: None Declared, J. K. Marshall Grant / Research support from: John K. Marshall has received honoraria from Janssen, AbbVie, Allergan, Bristol-Meyer-Squibb, Ferring, Janssen, Lilly, Lupin, Merck, Pfizer, Pharmascience, Roche, Shire, Takeda and Teva., S. Rangarajan: None Declared, S. Islam: None Declared, A. Bahonar: None Declared, K. F. Alhabib: None Declared, A. Kontsevaya: None Declared, F. Ariffin: None Declared, H. U. Co: None Declared, W. Al Sharief: None Declared, A. Szuba: None Declared, A. Wielgosz: None Declared, M. L. Diaz: None Declared, R. Yusuf: None Declared, L. Kruger: None Declared, B. Soman: None Declared, Y. Li: None Declared, C. Wang: None Declared, L. Yin: None Declared, M. Erkin: None Declared, F. Lanas: None Declared, K. Davletov: None Declared, A. Rosengren: None Declared, P. Lopez-Jaramillo: None Declared, R. Khatib: None Declared, A. Oguz: None Declared, R. Iqbal: None Declared, K. Yeates: None Declared, Á. Avezum: None Declared, W. Reinisch Consultant of: Speaker for Abbott Laboratories, Abbvie, Aesca, Aptalis, Astellas, Centocor, Celltrion, Danone Austria, Elan, Falk Pharma GmbH, Ferring, Immundiagnostik, Mitsubishi Tanabe Pharma Corporation, MSD, Otsuka, PDL, Pharmacosmos, PLS Education, Schering-Plough, Shire, Takeda, Therakos, Vifor, Yakult, Consultant for Abbott Laboratories, Abbvie, Aesca, Algernon, Amgen, AM Pharma, AMT, AOP Orphan, Arena Pharmaceuticals, Astellas, Astra Zeneca, Avaxia, Roland Berger GmBH, Bioclinica, Biogen IDEC, Boehringer-Ingelheim, Bristol-Myers Squibb, Cellerix, Chemocentryx, Celgene, Centocor, Celltrion, Covance, Danone Austria, DSM, Elan, Eli Lilly, Ernest & Young, Falk Pharma GmbH, Ferring, Galapagos, Genentech, Gilead, Grünenthal, ICON, Index Pharma, Inova, Janssen, Johnson & Johnson, Kyowa Hakko Kirin Pharma, Lipid Therapeutics, LivaNova, Mallinckrodt, Medahead, MedImmune, Millenium, Mitsubishi Tanabe Pharma Corporation, MSD, Nash Pharmaceuticals, Nestle, Nippon Kayaku, Novartis, Ocera, Omass, Otsuka, Parexel, PDL, Periconsulting, Pharmacosmos, Philip Morris Institute, Pfizer, Procter & Gamble, Prometheus, Protagonist, Provention, Robarts Clinical Trial, Sandoz, Schering-Plough, Second Genome, Seres Therapeutics, Setpointmedical, Sigmoid, Sublimity, Takeda, Therakos, Theravance, Tigenix, UCB, Vifor, Zealand, Zyngenia, and 4SC, Advisory board member for Abbott Laboratories, Abbvie, Aesca, Amgen, AM Pharma, Astellas, Astra Zeneca, Avaxia, Biogen IDEC, Boehringer-Ingelheim, Bristol-Myers Squibb, Cellerix, Chemocentryx, Celgene, Centocor, Celltrion, Danone Austria, DSM, Elan, Ferring, Galapagos, Genentech, Grünenthal, Inova, Janssen, Johnson & Johnson, Kyowa Hakko Kirin Pharma, Lipid Therapeutics, MedImmune, Millenium, Mitsubishi Tanabe Pharma Corporation, MSD, Nestle, Novartis, Ocera, Otsuka, PDL, Pharmacosmos, Pfizer, Procter & Gamble, Prometheus, Sandoz, Schering-Plough, Second Genome, Setpointmedical, Takeda, Therakos, Tigenix, UCB, Zealand, Zyngenia, and 4SC, P. Moayyedi: None Declared, S. Yusuf: None Declared

